# Light in Vacuum Tubes

**Published:** 1897-07

**Authors:** 


ARTICLE V.

### LIGHT IN VACUUM TUBES.

At the second day's session of the electricians' convention held at the Electrical Exposition, D. MacFarlan Moore lectured on "The Light of the Future."

The light of the future, according to Mr. Moore, is a glorified electric light, depending neither upon an incandescent filament in a glass bulb nor on a glowing crater between two carbon pencils. It is "nothing in a state of intense vibration," and the result is a brilliant purplish light radiating from long glass tubes.

Mr. Moore demonstrated the practicability of his invention—light without heat—before a large assembly.

Around the stage, suspended by wires from the ceiling, were a number of closed glass tubes, each about ten feet in length; each tube had been exhausted till only about one millionth of an atmosphere was left. By means of a small electrical device, the few atoms in the tube were set rapidly vibrating and the hall was completely illuminated by the light which issued from the tubes.

In explanation of the phenomenon of light by inductive effect in vacuum tubes, Mr. Moore described the methods he uses. The vacuum vibrator is the nucleus of his invention. It consists merely of a spring fixed at one end and free at the other. To the free end, a small disk of soft iron is attached, and the center of the spring rests against a contact point. All this is inclosed in a small exhausted tube. The only other apparatus is a small electro-magnet and a long glass tube. A current from the dynamo is passed through the coil of the magnet and then through the vibrator, the soft iron disk on the vibrator spring being placed above the magnet. The wires are attached to the two ends of the magnet coil and also to·the two ends of the long glass tube. When the circus through the magnet and the vibrator is closed, the small disk at the end of the spring vibrates with great rapidity and breaks the current in the vacuum with each vibration. This rapid vibration is communicated to the atoms in the long tube, and the result is a light.

Mr. Moore explained to the audience the different steps which led him so far toward the desired result, each step being illustrated by some piece of apparatus and the intensity of the light increasing with each step. The long tubes contain nothing, not even a wire, but each end is in contact with the two wires from the magnetic coils. This is all. And yet the tube becomes a rod of solid opalescent light, which shimmers and glows in the tube as the atoms oscillate under the influence of the rapidly disrupted current in the vibration. It will be remembered that on the opening of the exposition a frame of these tubes was so arranged in the gallery that Governor Morton, as he touched the key which set the machinery in motion, appeared framed in the beautiful etheric light, while above him glistened the words "Let there be light."

Mr. Moore is about twenty-seven years old, and up to two years ago worked at the draughting table of a large electrical concern. He is a graduate of Lehigh University, and for years has been interested in mechanics and electricity. It is not more than eighteen months since he first showed to a few skeptical friends a little tube of two inches, from the ends of which he connected two wires to the terminals of the socket of an ordinary electric fixture, causing the little tube to glow with the purplish light he had so intensified.—*N. Y. Tribune.*

